# Optimizing the target detectability of cone beam CT performed in image‐guided radiation therapy for patients of different body sizes

**DOI:** 10.1002/acm2.12306

**Published:** 2018-03-08

**Authors:** Ching‐Ching Yang, Pei‐Chieh Yu, Jau‐Ming Ruan, Yu‐Cheng Chen

**Affiliations:** ^1^ Department of Medical Imaging and Radiological Sciences Tzu‐Chi University of Science and Technology Hualien Taiwan; ^2^ Department of Radiation Oncology Cathay General Hospital Taipei Taiwan; ^3^ School of Medicine China Medical University Taichung Taiwan

**Keywords:** cone beam computed tomography, image‐guided radiation therapy, radiation dose, target detectability

## Abstract

**Purpose:**

The target detectability of cone beam computed tomography (CBCT) performed in image‐guided radiation therapy (IGRT) was investigated to achieve sufficient image quality for patient positioning over a course of treatment session while maintaining radiation exposure from CBCT imaging as low as reasonably achievable (ALARA).

**Methods:**

Body CBCT scans operated in half‐fan mode were acquired with three different protocols: CBCT_lowD_, CBCT_midD_, and CBCT_highD_, which resulted in weighted CT dose index (CTDI_w_) of 0.36, 1.43, and 2.78 cGy, respectively. An electron density phantom that is 18 cm in diameter was covered by four layers of 2.5‐cm‐thick bolus to simulate patients of different body sizes. Multivariate analysis was used to examine the impact of body size, radiation exposure, and tissue type on the target detectability of CBCT imaging, quantified as contrast‐to‐noise ratio (CNR).

**Results:**

CBCT_midD_ allows sufficient target detection of adipose, breast, muscle, liver in a background of water for normal‐weight adults with cross‐sectional diameter less than 28 cm, while CBCT_highD_ is suitable for adult patients with larger body sizes or body mass index over 25 kg/m^2^. Once the cross‐sectional diameter of adult patients is larger than 35 cm, the CTDI_w_ of CBCT scans should be higher than 2.78 cGy to achieve required CNR. As for pediatric and adolescent patients with cross‐sectional diameter less than 25 cm, CBCT_lowD_ is able to produce images with sufficient target detection.

**Conclusion:**

The target detectability of soft tissues in default CBCT scans may not be sufficient for overweight or obese adults. Contrary, pediatric and adolescent patients would receive unnecessarily high radiation exposure from default CBCT scans. Therefore, the selection of acquisition parameters for CBCT scans optimized according to patient body size was proposed to ensure sufficient image quality for daily patient positioning in radiation therapy while achieving the ALARA principle.

## 1 INTRODUCTION

Approximately 50% of cancer patients can benefit from radiation therapy in the management of their diseases.^1^, ^2^, ^3^ The accuracy of radiation therapy depends on the conformal deposition of ionizing radiation to the target volume and the efforts to spare its neighboring healthy tissues.^4^, ^5^ To achieve high‐precision treatments, imaging plays a crucial role in planning and delivering radiation beams.^6^, ^7^ It has been demonstrated that the use of image‐guided radiation therapy (IGRT) may improve the clinical outcome of patients undergoing radiation therapy.^8^, ^9^ In our department, patients are routinely scanned by multidetector computed tomography (MDCT) scanners for planning purposes before treatment. During daily treatment, cone beam CT (CBCT) mounted on the gantry of linear accelerator is used to detect target position relative to the planned radiation beams to improve the accuracy of treatment delivery through geometric corrections. Target detectability of CBCT is a very important image quality metric to achieve a high level of patient positioning and treatment accuracy.^10^, ^11^, ^12^, ^13^ In spite of the increasing use of CBCT to verify and correct patient setup, the contrast resolution of CBCT in delineating soft tissue structures is lower than that of MDCT.^14^, ^15^ MDCT has approximately 3 Hounsfield units (HU) contrast resolution, while CBCT allows a contrast resolution of 10 HU.^16^ CBCT imaging performed in IGRT is usually acquired by using particular imaging geometry, beam characteristics, and reconstruction method for a specific body part in clinical routine practice. However, individual body dimensions would affect the photon statistics in CBCT data, where patients of larger body sizes receive reduced radiation to the isocenter and internal organs, thus causing degradation in image quality.^17^ These characteristics of CBCT imaging indicate that optimizing the scan protocols according to patient dimensions is essential to ensure sufficient image quality for daily patient positioning in radiation therapy. Fractionated radiation treatments are usually delivered in 20 fractions to improve patient tolerance, so the total radiation dose from CBCT is a factor of 20 greater than that of a single scan.^18^ Besides, CBCT doses are distributed to the entire imaging region, not only the target volume.^19^ Hence, it is necessary to know what the radiation doses are from CBCT and raise awareness of using lower radiation doses. In this study, the tradeoff between target detectability and radiation dose was investigated for CBCT performed in IGRT to achieve sufficient image quality for patient positioning over a course of treatment session while maintaining radiation exposure as low as reasonably achievable (ALARA). The performance of routine MDCT in target detection was also evaluated for comparison purpose.

## 2 MATERIALS AND METHODS

### 2.A. MDCT and CBCT scans

All MDCT scans were performed using a GE Discovery CT590 RT CT simulator (GE Healthcare, Waukesha, WI, USA). The CT simulator is a 16‐slice MDCT with 16 × 1.25 mm collimation. For the automatic exposure control (AEC) system (AutomA 3D; GE Healthcare, Waukesha, WI, USA) on the MDCT scanner we operate, the parameter used to specify the desired image quality is the noise index (NI), which is approximately equal to the standard deviation of HUs in the central region of a homogeneous phantom image. Moreover, the AEC system allows the operator to define the mA range (minimum to maximum mA) within which the tube current can be modulated. The vendor default settings for routine MDCT body scans were used in this study, including 120 kVp, NI of 12, mA range of 100–440 mA, pitch of 1.375, and gantry rotation time of 0.92 s. The volume CT dose index (CTDI_vol_) reported by the scanner console was recorded in a DICOM dose report file after each scan. The MDCT images were reconstructed by adaptive statistical iterative reconstruction (ASIR) 40% in slice mode (SS40) with matrix size of 512 × 512 and voxel size of 1.04 × 1.04 × 2.5 mm^3^.

CBCT images were acquired using the on‐board imager system installed on a Varian TrueBeam STX radiation therapy machine (Varian Medical System, Palo Alto, CA, USA). Preset parameters are configured per anatomical site for imaging geometry, beam characteristics, and reconstruction method. In the CBCT system we operate, body scans can be obtained in three vendor default modes: Thorax (CBCT_lowD_), Pelvis (CBCT_midD_), and Pelvis Obese (CBCT_highD_) (Table 1). Once a specific CBCT mode was chosen, the corresponding weighted CT dose index (CTDI_w_) was displayed on the operator's console prior to image acquisition. The CBCT_lowD_ protocol used 125 kVp and 270 mAs, which resulted in CTDI_w_ of 0.36 cGy. The CBCT_midD_ protocol used 125 kVp and 1080 mAs, while the CBCT_highD_ protocol used 140 kVp and 1687.5 mAs. The CTDI_w_ from CBCT_midD_ and CBCT_highD_ are 1.43 cGy and 2.78 cGy, respectively. For these body scans, CBCT was operated in half‐fan mode to cover field of view (FOV) that is 46.5 cm in diameter and 18 cm in length. The CBCT images were reconstructed with matrix size of 512 × 512 and voxel size of 0.908 × 0.908 × 1.989 mm^3^ using the Feldkamp–Davis–Kress (FDK) algorithm with standard reconstruction filter and medium ring artifact suppression algorithm.

**Table 1:** The acquisition parameters of CBCT scans and the resulting radiation dose

	Tube voltage	Tube current‐time product	CTDI_w_
CBCT_lowD_	125 kVp	270 mAs (20 mA, 13.5 s)	0.36 cGy
CBCT_midD_	125 kVp	1080 mAs (80 mA, 13.5 s)	1.43 cGy
CBCT_highD_	140 kVp	1687.5 mAs (100 mA, 16.9 s)	2.78 cGy

### 2.B Image quality evaluation

**Figure 1:**
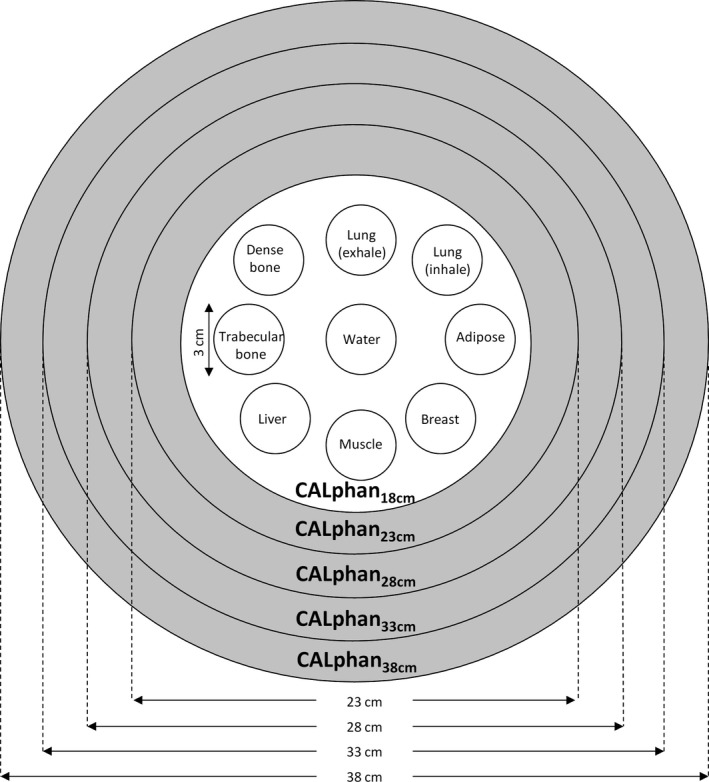
Illustration of the calibration phantoms simulating patients of different body sizes.

The calibration phantoms consisting of an electron density phantom and additional annuluses were used to evaluate the image quality of CBCT performed in IGRT (Fig. 1). The electron density phantom (Model 062; CIRS, Norfolk, VA, USA) which is 18 cm in diameter and 5 cm in height was covered by 4 layers of 2.5‐cm‐thick bolus (Superflab Bolus; Radiation Products Design Inc, Albertville, MN, USA) to enlarge the diameter of the calibration phantom from 18 cm (CALphan_18 cm_) to 23 cm (CALphan_23 cm_), 28 cm (CALphan_28 cm_), 33 cm (CALphan_33 cm_), and 38 cm (CALphan_38 cm_). The electron density phantom is made of soft tissue equivalent epoxy resin with nine rod inserts simulating lung (inhale: 0.195 g/cc; exhale: 0.51 g/cc), adipose (0.96 g/cc), breast (0.991 g/cc), plastic water (1.016 g/cc), muscle (1.062 g/cc), liver (1.072 g/cc), trabecular bone (1.161 g/cc), and dense bone (1.53 g/cc). The nested disk of the electron density phantom was made from plastic water (1.016 g/cc). The contrast‐to‐noise ratio (CNR) is an important index for the detection and diagnosis of structure and details of interest in CT,^20^ so CNR was used in this study to quantify the target detectability in both MDCT and CBCT. A circular region‐of‐interest (ROI) of 31 pixels was placed on the target and background regions in 11 slices (the central slice ±5 slices) to calculate the mean and standard deviation of HUs within ROI. The target ROIs were located at the rod inserts simulating various tissue materials, while the background ROI was located at the rod insert made of plastic water. The CNR was defined as

(1) $$\mathrm{CNR}=\frac{\left|\mathrm{CT}\#-\mathrm{CT}{\#}_{\mathrm{BG}}\right|}{SD_{\mathrm{BG}}}$$

where CT# is the mean CT number of the target region, CT#_BG_ and SD_BG_ are the average and standard deviation of CT numbers of the background region, respectively. A CNR of 1.0 occurs when the image contrast (or difference) between target and background was equal to the background noise. Based on our clinical experiences, a CNR of 5 is required for target detection perceived by naked eyes to ensure sufficient geometric accuracy.

### 2.C Multivariate analysis

There is a well‐recognized tradeoff between image quality and radiation dose in CT imaging.^21^, ^22^, ^23^, ^24^, ^25^, ^26^, ^27^ In our department, the tube current of routine MDCT body scans is modulated automatically by the AEC system to achieve consistent image quality for different patient sizes or body parts. On the other hand, the scan mode of CBCT imaging configured per anatomical site is selected based on operator experience to compensate for variations in individual body dimensions. Although the manual selection of CBCT scan mode also depends on patient body size, no straightforward relationship between the image quality degradation and the variations in patient dimensions have been established to ensure sufficient image quality for patient positioning. Besides body size, there are other factors that might affect the image quality of CBCT, such as scanner design, tube current, tube voltage, scan time, and so on. Hence, multiple linear regression methods were used to assess how patient dimension, radiation dose and tissue type affect the image quality of CBCT for patient positioning in radiation therapy, quantified by CNR. These influencing factors were chosen to model the overall physical and biological processes involved in CBCT scans. A similar idea has been proposed by Brambilla et al. to investigate the impact of various factors on the image quality of positron emission tomography (PET).^28^ Our model to explain the relationship between independent and dependent variables was:

(2) $$\begin{array}{cc} \mathrm{CNR}\;\; & =\;B_{0}+B_{1}\times (\frac{1}{\mathrm{bodysize}})+B_{2}\times \left(\mathrm{CTD}I_{w}\right)\\ & \;+B_{3}\times (|\exp(\mathrm{density})-\exp(1.016)|) \end{array}$$

where B_0_ to B_3_ are the regression coefficient (B) to be estimated. The body size is the diameter of the calibration phantoms. The exp(density) is the exponential of the density for the rod inserts in the calibration phantoms, where 1.016 g/cc is the density of plastic water. The standard regression coefficient (β) was calculated to assess the relative importance of each predictor. Student's t test and variance inflation factor (VIF) were used as criteria in screening the potential regression model. A predictor was considered statistically significant if |t| >2. A maximal VIF value in excess of 10 was regarded as an indication that multicollinearity may be unduly influencing the least square estimates. The coefficient of determination (R^2^) was calculated to assess the strength of the functional regression model. The statistical analysis algorithms were implemented in MATLAB 7.1 (The Mathworks, Natick, MA, USA).

## 3 RESULTS

**Figure 2:**
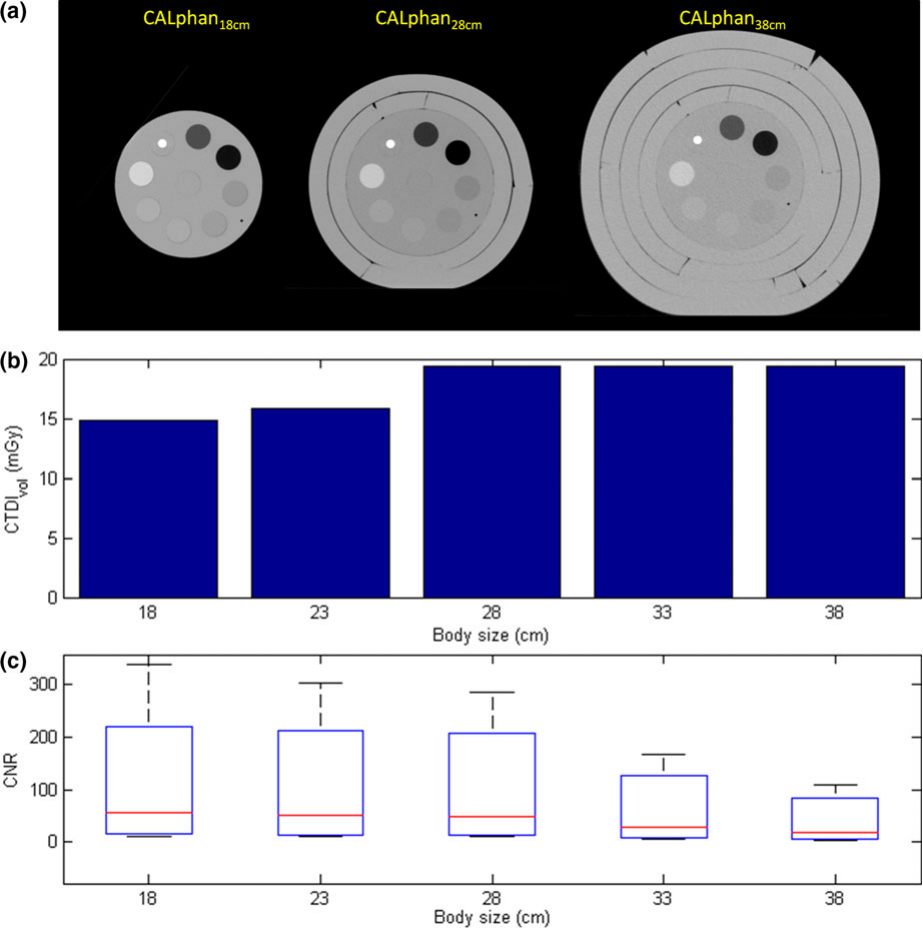
(a) Axial images, (b) CTDI_vol_, and (c) CNR of MDCT images acquired with the calibration phantoms simulating patients of different body sizes.

**Figure 3:**
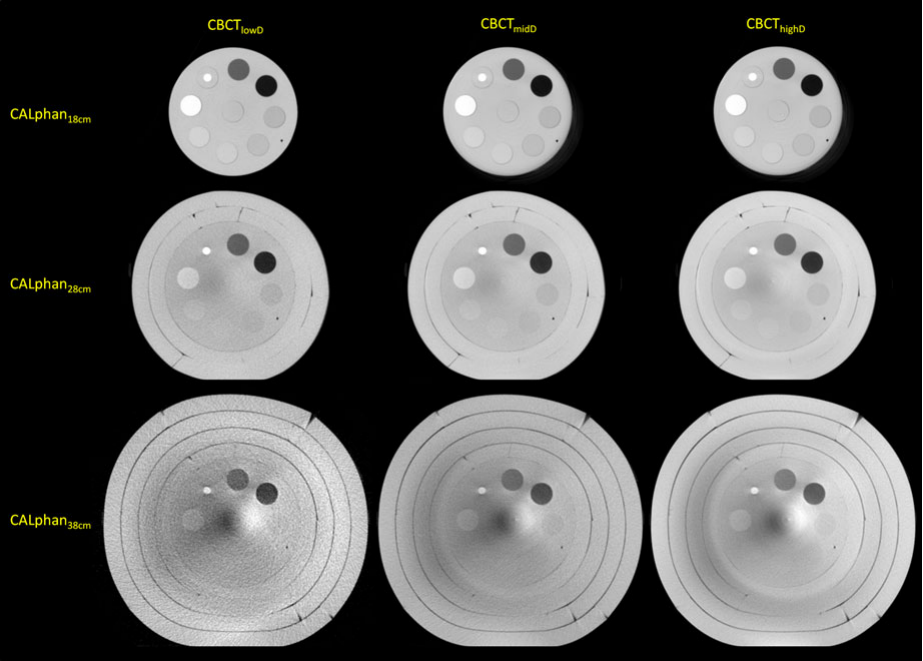
Axial images acquired with CBCT_lowD_, CBCT_midD_, and CBCT_highD_ (left to right) for CALphan_18 cm_, CALphan_28 cm_, and CALphan_38 cm_ (top to bottom).

**Figure 4:**
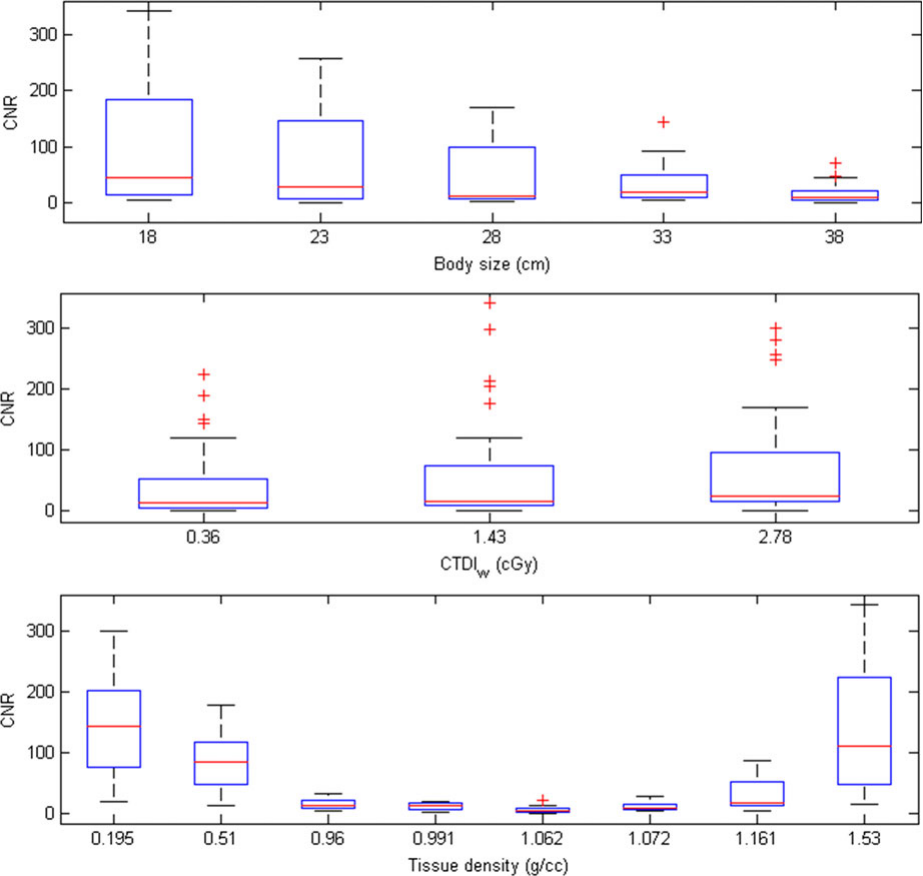
Box and whisker diagrams for CNR of CBCT images with respective to (a) body size, (b) CTDI_w_, and (c) tissue density.

Since the AEC system was activated when performing MDCT body scans, the tube current was varied according to the phantom sizes. The mean values of tube current in MDCT scans were 324, 340, 440, 440, 440 mA for CALphan_18 cm_, CALphan_23 cm_, CALphan_28 cm_, CALphan_33 cm_, and CALphan_38 cm_, respectively. Figure 2(a) shows the axial images of MDCT for CALphantom_18 cm_, CALphantom_28 cm_, CALphantom_38 cm_ (width, 400 HU; level, 40 HU). Figure 2(b) and 2(c) demonstrate the CTDI_vol_ and CNR of MDCT images acquired with all five calibration phantoms, respectively. For the box and whisker plot shown in Fig. 2(c), the red line in each box represents the median of the distribution, whereas the top and bottom of each box represent the 25^th^ and 75^th^ percentile of the distribution, respectively. The whiskers extend to the 99.3% confidence interval (±2.7 sigma). As seen in Fig. 2(c), substantial decrease in CNR was found in CALphan_33 cm_ and CALphan_38 cm_. These results indicate that AEC compensates for the increase in photon attenuation by increasing the tube current until reaching the maximum value that the scanner can provide, i.e., 440 mA. Beyond the proportional limit that the AEC system can operate, the target detectability of MDCT was degraded in phantoms of larger sizes. Figure 3 shows the axial images acquired using CBCT_lowD_, CBCT_midD_, CBCT_highD_ (left to right) for CALphan_18 cm_, CALphan_28 cm_, and CALphan_38 cm_ (top to bottom). The shading artifacts were not seen in CALphan_18 cm_, but became more pronounced in larger phantoms. For the same calibration phantom, the pattern of shading artifacts was similar in CBCT images acquired with different protocols. The box and whisker plots shown in Fig. 4 summarize the impact of body size, CTDI_w_ and tissue density on CNR of CBCT imaging performed in IGRT. For these box and whisker plots, any value outside the whiskers is considered to be an outlier and marked with a red cross. All data in Fig. 4 were used for multivariate analysis (Eq. (2)) to avoid distortion from the exclusion of genuine outliers. The results of regression analysis for CBCT images of the calibration phantoms acquired with three scan modes are shown in Table 2. The regression equation that expresses the relationship between CNR and the predictors for CBCT performed in our department is:

(3) $$\begin{array}{cc} \mathrm{CNR}\;\; & =-5.12+167.93\times (\frac{1}{\mathrm{bodysize}})+0.81\times \left(\mathrm{CTD}I_{w}\right)\\ & \;\;\;+5.11\times \left(|\exp(\mathrm{density})-\exp(1.016)|\right) \end{array}$$

**Table 2:** Statistical analysis results of the regression model for phantom studies (R^2^ = 0.8026)

Predictor	B	β	t^a^	VIF^b^
1/body size	167.93	0.40	9.65	1.00
CTDI_w_	0.81	0.18	4.33	1.00
|exp(density) – exp(1.016)|	5.11	0.78	18.97	1.00

a A predictor is considered to be statistically significant if |t| >2.

b A maximum VIF value in excess of 10 is taken as an indication that multicollinearity may be unduly influencing the least square estimates.

**Figure 5:**
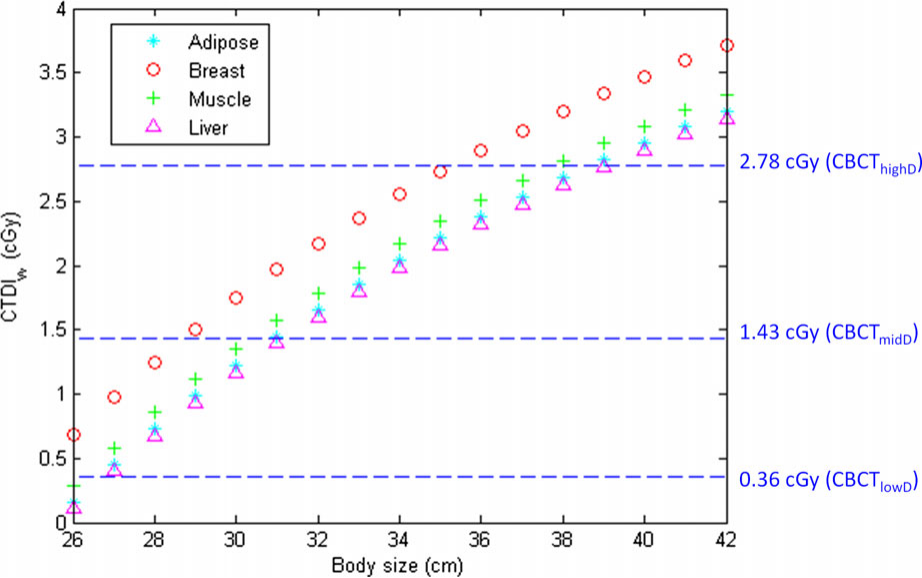
CTDI_w_ estimated based on the regression model to achieve CNR = 5 for patients of different body sizes.

The regression model in Eq. (3) yielded an R^2^ of 0.8026. Figure 5 shows the CTDI_w_ required for CBCT images achieving CNR = 5 as a function of body size estimated based on the regression model in Eq. (3) for four different types of soft tissues. The dashed blue lines indicate the CTDI_w_ of CBCT_highD_ (top), CBCT_midD_ (mid), and CBCT_lowD_ (bottom).

## 4 DISCUSSION

Based on naked‐eye observation of Fig. 3, the discrimination of rod inserts from the background region becomes more difficult for CBCT images acquired with larger calibration phantoms. This phenomenon was also verified quantitatively in Fig. 4(a). The degradation of target detectability in larger phantoms may be owing to (1) the increase of scattered radiation and (2) the decrease of photon flux. Compared with MDCT, CBCT imaging contains a larger amount of scattered radiation mainly due to a larger FOV of cone beam geometry. Scatter is a very important artifact causing factor in CBCT. The scatter‐to‐primary ratio (SPR) is about 0.01 for single‐ray CT and 0.05–0.15 for fan‐beam and spiral CT, and may be as large as 0.4–2.0 in CBCT.^10^, ^11^, ^12^, ^13^, ^29^, ^30^, ^31^ Typical scatter artifacts show as shading or streaks, which would result in reduced contrast resolution and increased noise in CBCT imaging. Several scatter correction algorithms have been proposed to compensate for the shading artifacts in CBCT, but there is no uniformly accepted solution yet.^30^, ^31^ Because of the heavier attenuation caused by larger calibration phantoms, the statistical fluctuations in their CBCT imaging become greater. Consequently, the increased image noise due to quantum mottle would degrade the contrast resolution of CBCT and lead to the loss of geometric information for patient positioning. Hence, when CBCT images were acquired with higher tube voltage or tube current, the target detectability was improved by reducing the quantum fluctuations in CBCT, which was verified qualitatively in Fig. 3 and quantitatively in Fig. 4(b). As seen in Fig. 3, the rod inserts simulating bone and lung can still be differentiated from the background region when CALphan_38 cm_ was imaged by CBCT_lowD_, but not for the rod inserts simulating soft tissues. Moreover, it was found in Fig. 4(c) that the rod inserts simulating bone and lung have higher CNR values. These findings indicate that the selection of acquisition parameters and the variation of patient dimensions would influence the detectability of soft tissues in CBCT more seriously.

Although the target detectability of CBCT performed in IGRT can be improved by using higher tube voltage or tube current, the radiation exposure to patients is also increased. Compared with adults, children are more radiosensitive and have longer postirradiation life expectancy. According to the National Academies of Sciences Biologic Effects of Ionizing Radiation (BEIR) VII report, the radiation‐induced lifetime attributable risk (LAR) of all forms of cancer from 100 mSv are 1.445%, 1.816%, and 2.414% for 10‐, 5‐, and 1‐year‐old males, respectively, and are 2.611%, 3.377%, and 4.479% for 10‐, 5‐, and 1‐year‐old females, respectively.^32^ Thus, CT scan parameters should be optimized to ensure sufficient image quality while achieving the ALARA principle. Practically every MDCT system is delivered with AEC system nowadays, no matter it is a stand‐alone scanner or a hybrid scanner. Hence, protocol optimization for patients of different body sizes can be achieved automatically during data acquisition in MDCT imaging with the use of AEC technique. On the other hand, the CBCT imaging performed in IGRT is often acquired with particular scan modes configured per anatomical site for imaging geometry, beam characteristics, and reconstruction method, and the default settings are commonly designed for normal‐weight adults. Hence, the image quality of CBCT acquired with these scan modes may not be sufficient for overweight or obese adult patients. Contrary, pediatric and adolescent patients may receive unnecessarily high radiation exposure during CBCT scans that were performed with scan modes designed for adults. In order to tailor the scan protocols of CBCT imaging performed in IGRT according to patient body size, it is important to understand the impact of body size and radiation exposure on the image quality of CBCT for achieving a high level of patient positioning and treatment accuracy in radiation therapy. Hence, multivariate analysis was used to examine the CNR from phantom studies acquired under various imaging conditions. Since tissue type is also an important influencing factor of target detection, it was also included in the model.

The regression relationship has R^2^ larger than 0.80, indicating a good fit to the data. According to our results in Table 2, it was found that all independent variables are statistically significant predictors of CNR (|t| > 2), whereas $|\exp\left(\mathrm{density}\right)-\exp\left(1.016\right)|$ is the most significant predictor (β = 0.78), followed by 1/body size (β = 0.40) and CTDI_w_ (β = 0.18). High multicollinearity was not observed among independent variables in the model (VIF <10). The CTDI_w_ as a function of body size, estimated based on the regression model in Eq. (3), was proposed to ensure sufficient detection of soft tissues for CBCT performed in IGRT (Fig. 5). It was found that the scan protocols of CBCT_midD_ and CBCT_highD_ could be used to image patients with cross‐sectional diameter less than 28 and 35 cm, respectively. For the abdomen and pelvis of an anthropomorphic phantom simulating normal‐weight adults (Rando; Alderson Research Laboratories, Standford, CT, USA), the square roots of the product of long‐axis and short‐axis diameter are 23 and 26 cm, respectively. The corresponding results for an anthropomorphic phantom simulating 10‐year‐old children (ATOM; CIRS, Norfolk, VA, USA) are 17 and 19 cm. Therefore, CBCT_midD_ may allow sufficient image quality in abdominal and pelvic scans for normal‐weight adults with cross‐sectional diameter less than 28 cm, while CBCT_highD_ could be suitable for adult patients in other body status (i.e., normal‐weight adults with cross‐sectional diameter larger than 28 cm, overweight and obese adults). Once the cross‐sectional diameter of adult patients is larger than 35 cm, the CTDI_w_ from CBCT scans should be higher than 2.78 cGy to achieve CNR of 5. As for pediatric and adolescent patients with cross‐sectional diameter less than 25 cm, CBCT_lowD_ is able to produce images with sufficient target detection in abdominal and pelvic scans. Several limitations to this study need to be acknowledged. First, the calibration phantoms are meant to simulate patients with specific body shape and tissue composition, which limit generalization of the results to a population of heterogeneous body types. Second, the ROI selection and the choice of image quality metrics have a large impact on the definition of optimal scan protocol. This study aimed to optimize the target detectability of on‐board CBCT in radiation therapy to achieve a high level of patient positioning and treatment accuracy, so a task‐specific optimization based on multivariate analysis was performed. Third, the coefficients of the multivariate model in Eq. (3) represent the overall physical and biological processes involved in CBCT scans performed in our routine practice, so a different model should be built once the imaging geometry, beam characteristics, or reconstruction method is changed. Last, only a single‐manufacturer's CBCT system performed in IGRT was investigated, so the optimized protocols cannot be applied to other imaging systems from different manufacturers. Additional studies assessing the proposed optimization workflow for different image‐guided systems used in IGRT will be needed and valuable.

## 5 CONCLUSION

The tradeoff between target detectability and radiation dose was investigated for CBCT performed in IGRT to ensure sufficient image quality for daily patient positioning in radiation therapy while achieving the ALARA principle. Multivariate analysis was used to examine the impact of body size, radiation exposure and tissue type on the target detectability of CBCT imaging, quantified by CNR. Based on our results, CBCT_midD_ is able to produce images with sufficient target detection of adipose, breast, muscle, and liver in a background of water for normal‐weight adults with cross‐sectional diameter less than 28 cm, while CBCT_highD_ should be used for adult patients with larger body sizes or higher body mass index. Once the cross‐sectional diameter of adult patients is larger than 35 cm, the CTDI_w_ of CBCT scans needs to be higher than 2.78 cGy to achieve CNR of 5. As for pediatric and adolescent patients with cross‐sectional diameter less than 25 cm, CBCT_lowD_ may allow sufficient image quality in abdominal and pelvic scans.

## CONFLICT OF INTEREST

The authors have no relevant conflicts of interest to disclose.
